# Prediction of difficulty in cryoballoon ablation with a three‐dimensional deep learning model using polygonal mesh representation

**DOI:** 10.1002/joa3.70078

**Published:** 2025-04-25

**Authors:** Kazutaka Nakasone, Makoto Nishimori, Masakazu Shinohara, Kunihiko Kiuchi, Mitsuru Takami, Kimitake Imamura, Yu Izawa, Toshihiro Nakamura, Yusuke Sonoda, Hiroyuki Takahara, Kyoko Yamamoto, Yuya Suzuki, Kenichi Tani, Hidehiro Iwai, Yusuke Nakanishi, Ken‐ichi Hirata, Koji Fukuzawa

**Affiliations:** ^1^ Division of Cardiovascular Medicine, Department of Internal Medicine Kobe University Graduate School of Medicine Kobe Hyogo Japan; ^2^ Division of Molecular Epidemiology Kobe University Graduate School of Medicine Kobe Hyogo Japan; ^3^ Section of Arrhythmia, Division of Cardiovascular Medicine, Department of Internal Medicine Kobe University Graduate School of Medicine Kobe Hyogo Japan

**Keywords:** artificial intelligence, atrial fibrillation, computed tomography, cryoballoon ablation, deep learning

## Abstract

**Background:**

Cryoballoon ablation (CBA) is useful for pulmonary vein (PV) isolation. However, some cases are challenging, requiring multiple applications and/or touch‐up ablations. Although several predictors of CBA difficulty have been reported, none have assessed the spatial location and morphology of the left atrium and PVs. This study aimed to develop a three‐dimensional (3D) deep learning (DL) model to predict CBA difficulty and compare its accuracy with conventional manual measurement.

**Methods:**

A 28‐mm cryoballoon (Arctic Front Advance, Medtronic) was used in all cases. CBA difficulty was defined as requiring touch‐up ablation and/or more than three applications per PV. We developed a DL model that can learn polygonal meshes and predict CBA difficulty. In the conventional method, predictors included a thinner left lateral ridge, higher left superior PV (LSPV) ovality index, longer LSPV ostium‐bifurcation distance, and shorter right inferior PV ostium‐bifurcation distance.

**Results:**

A total of 189 patients who underwent CBA for drug‐resistant atrial fibrillation between January 2015 and January 2022 were included. The DL model was superior to the conventional method in accuracy (0.793 vs. 0.630, *p* = .042) and specificity (0.796 vs. 0.609, *p* = .022), with the AUC‐ROC of 0.821.

**Conclusions:**

We developed a 3D DL model that can detect CBA difficulty using a polygonal mesh representation. By predicting difficult cases in advance, strategies can be developed to increase success rates.

## Graphical abstract

Three‐dimensional (3D) geometries reconstructed from computed tomography (CT) images were utilized as input for a deep learning model to classify the cryoballoon ablation (CBA) difficulty. The AUC‐ROC estimated by bootstrapping was 0.821 (95% CI: 0.646–0.995), indicating the potential effectiveness of this novel deeplearning‐based predictive approach.

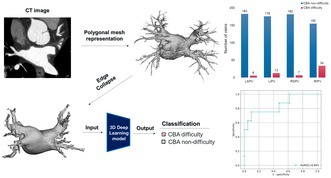

## 1 INTRODUCTION

Pulmonary vein isolation (PVI) has become the gold standard for catheter ablation of atrial fibrillation (AF).^1^ Cryoballoon ablation (CBA) is simpler than PVI using radiofrequency catheter ablation (RFCA) and has fewer critical complications, and has been increasingly indicated for AF ablation in recent years.^2^, ^3^ However, it is sometimes difficult to occlude the left atrial (LA)—pulmonary veins (PV) border with a balloon because the border is not always round in shape, leaving a conduction gap that requires additional procedures with RFCA in 3%–23% of cases.^4^, ^5^, ^6^ Simultaneous RFCA with CBA in one session is controversial because it prolongs the procedure time and increases medical costs.

Manual analysis of preoperative cardiac computed tomography (CT) images has been reported to evaluate the PV length, vascular ovality, and angle of each PV as a method for predicting the CBA difficulty before the procedure.^4^, ^5^, ^6^, ^7^, ^8^, ^9^, ^10^ However, these methods are complicated and have not been widely applied clinically; hence, automation is desirable.

Recently, artificial intelligence (AI) has been widely used in the medical and healthcare fields for diagnosis, prognosis, and risk prediction. AI can automatically extract features from the given data. Furthermore, several deep learning (DL) models have reported the use of cardiac CT images to predict and assess arrhythmia triggers in patients with paroxysmal AF and risk of left atrial appendage thrombosis, respectively.^11^, ^12^ However, these models have limitations; they either learn from two‐dimensional slices of cardiac CTs or include extracardiac structures, thereby reducing learning efficiency. Moreover, critical three‐dimensional (3D) spatial information about the heart is lost. Therefore, there is a need for methods that preserve and learn from 3D data.

Various 3D representation techniques exist, such as voxels, point clouds, and polygonal meshes.^13^, ^14^, ^15^ The voxel method directly incorporates CT images and handles its spatial values as digital data. This comprehensive approach includes extraneous information and requires a large dataset for DL model development. Next, the point‐cloud method represents the surface of objects as a collection of points; however, each point exists independently without relational information, making it challenging to distinguish the boundaries between the LA and PVs. In contrast, a polygon mesh represents the surface of objects using a polyhedron and expresses 3D structures using only points, edges, and faces. To the best of our knowledge, no study has used polygonal meshes to create DL models in the cardiovascular field. Therefore, the purpose of this study was to create a 3D DL model of the LA–PV from CT images of the heart using a polygon mesh representation to predict CBA difficulty and to compare its accuracy with that of the conventional method of manually measuring CT images.

## 2 METHODS

### 2.1 Study population

We retrospectively evaluated 247 patients who underwent CBA for AF between January 2015 and December 2021. The analysis was conducted on 189 patients, after excluding those without preoperative contrast‐enhanced CT images. All methods were performed following the relevant directives and regulations, including the Declaration of Helsinki, and informed consent was obtained from all participants. This clinical study was approved by the Ethical Review Board of the Kobe University Medical Ethical Committee (No. B220174) on January 5, 2023.

### 2.2 Mapping and ablation procedure

Our standard CBA has been previously reported in detail.^16^ Briefly, patients were studied under deep sedation while breathing spontaneously. Transseptal puncture was performed in all cases under intracardiac echocardiography (ICE) guidance using the brockenbrough technique. During the entire procedure, the activated clotting time was maintained for >350 s by supplementation with heparin infusion as required. Internal electrical cardioversion was performed to restore sinus rhythm if AF occurred. In addition, mapping and ablation were performed using a NavX system (Abbott, Chicago, IL, USA) as a guide. This was performed after the integration of a 3D model of the anatomy of the LA and PVs obtained from preoperative CT images. Before ablation, the circular mapping catheter (Optima; Abbott) and the ablation catheter‐reconstructed LA posterior anatomies were aligned with the CT image. PVI was performed using second‐generation cryoballoon (Arctic Front Advance; Medtronic, Minneapolis, MN, USA) with a freeze cycle of 180–240 s. If Time‐to‐Isolation was achieved within 60 s and the temperature dropped below −40°C, the freeze time was reduced to 180 s. After ablation, 3D electroanatomical mapping was performed. If local potentials remained in the PVs or carina, touch‐up RFCA was performed. Furthermore, esophageal temperature was monitored using an esophageal temperature monitoring system (SensiTherm Multi; Abbott) to avoid damage to it. If this temperature reached 20°C, the cryothermal application was immediately stopped. Standard diaphragmatic stimulation was performed using high‐output phrenic nerve pacing (20 mA, 1200 ms) during right PV ablation to avoid phrenic nerve injury (PNI). However, in the case of diminished/lost pacing capture, cryoapplication was immediately stopped. Therefore, applications that were interrupted early because of esophageal temperature or PNI were excluded from the analysis. CBA difficulty was defined as those that required touch‐up ablation and/or more than 3 times per pulmonary vein. Based on the procedural results, cases were classified into the CBA difficulty group and the CBA nondifficulty group. The procedures were performed by three experienced operators with more than 10 years of ablation experience or by eight operators with 2–5 years of experience under the supervision of an operator with more than 10 years of experience. The outcomes were also analyzed based on the operators' years of experience.

### 2.3 Manual analysis method

The analysis of the cardiac CT images was performed using Ziostation software (Ziosoft, Tokyo, Japan). The cardiac CT images were initially acquired in the axial, coronal, and coronal oblique planes. The PV ostium was defined as the point of inflection between the LA and PV walls. A plane perpendicular to the ostium was established using a reformatting technique on an adaptive plane and was used to measure the maximal and minimal diameters. The anatomical parameters measured on volume rendering included the length of the left superior PV (LSPV) and right inferior PV (RIPV) trunk from the ostium to the bifurcation and the thickness of the left lateral appendage. The ovality index of the PV ostia was calculated using the formula: 2 × (maximal diameter − minimal diameter) (maximal diameter + minimal diameter). Subsequently, the CT images were retrospectively analyzed by two observers, and any inconsistencies related to anatomical variants and measurements between the observers were resolved by discussion.

### 2.4 Conventional method

**FIGURE 1:**
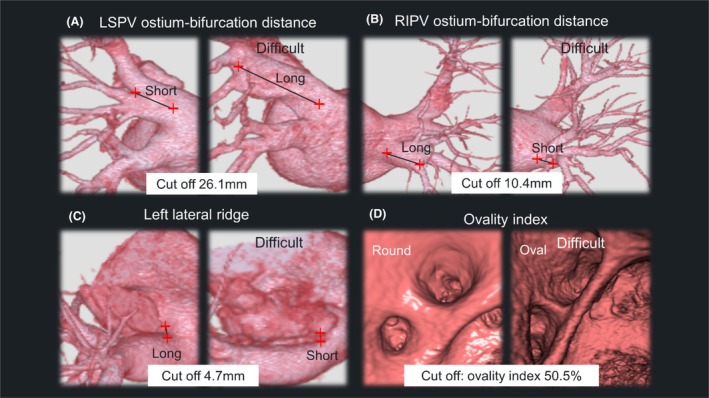
Conventional method for predicting the cryoballoon ablation difficulty. (A) a longer left superior pulmonary vein (LSPV) ostium‐bifurcation distance (>26.1 mm) (B) a shorter right inferior pulmonary vein (RIPV) ostium‐bifurcation distance (<10.4 mm) (C) a thinner left lateral ridge (<4.7 mm) (D) a higher ovality index of the LSPV (>50.5%).

We used the conventional model reported by Kajiyama et al. to compare the performance of the conventional model.^5^ In this method, a thinner left lateral ridge (<4.7 mm), a higher ovality index of the LSPV (>50.5%), a longer LSPV ostium‐bifurcation distance (>26.1 mm), and a shorter RIPV ostium‐bifurcation distance (<10.4 mm) were predictive factors requiring multiple applications. In the conventional method, “difficult” was defined as any case that met at least one of the four previously reported algorithms (Figure 1).

### 2.5 Data preprocessing

**FIGURE 2:**
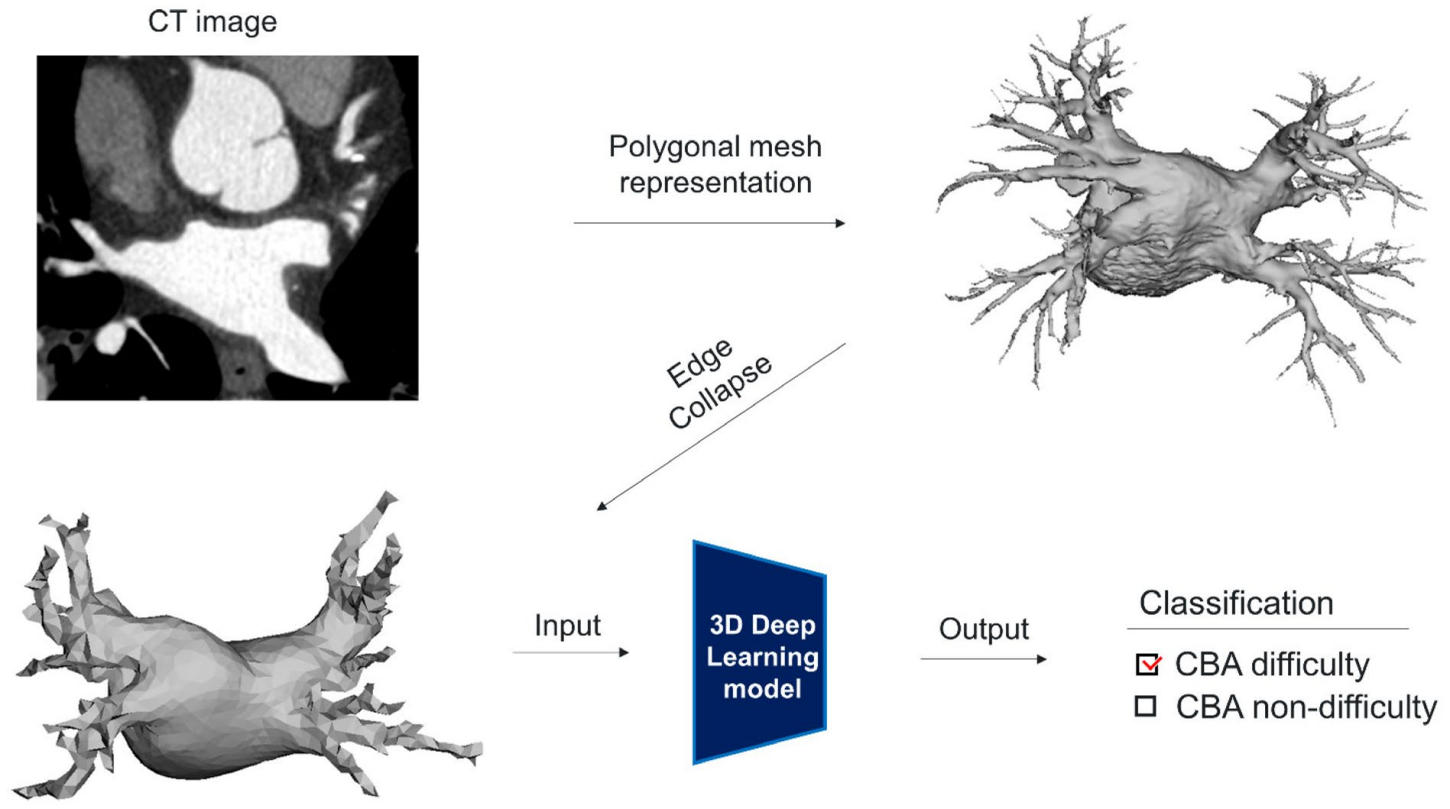
The overview of the deep learning architecture. Computed tomography (CT) images were extracted into a simplified data format that represents only the three‐dimensional (3D) geometry, and the data size was reduced through edge collapse. These data were input into the 3D deep learning model, which output binary data indicating either cryoballoon ablation (CBA) difficulty or CBA nondifficulty. CBA difficulty was defined as those that required touch‐up ablation and/or more than 3 times per pulmonary vein.

The conversion of cardiac contrast‐enhanced digital imaging and communications in medicine CT data into a polygon mesh format involves several stages. We began with using the Ziostation software (Ziosoft, Tokyo, Japan) to perform volumetric rendering. This step was crucial for isolating the LA and PV for focused analysis. Once the volumetric rendering was completed, we exported the CT images into a simplified data format that represented only the 3D geometry. This was achieved using the open‐source medical image processing software, Slicer3D, which allowed data output in the obj file format, which is the standard for representing 3D geometry (illustrated in Figure 2, top‐right).^17^ We used the Python library (PymeshLab) to reduce the data size for each sample.^18^ This library enabled us to perform edge collapse iteratively, ensuring that the number of vertices in each mesh did not exceed 400 (Figure 2, bottom left). This step was critical for optimizing the data for efficient processing and analysis in the subsequent AI model development. By leveraging these advanced software tools and techniques, we were able to transform complex CT data precisely and efficiently into a more manageable and analyzable polygon mesh format, thereby establishing the stage for the development of our innovative AI model.

### 2.6 Deep learning model

For the analysis, the complete dataset was stratified into two primary segments: 15% was allocated as the test dataset, and the remaining 85% was divided into training and validation datasets. This 85% was subjected to 5‐fold cross‐validation to enhance model robustness and reliability. In addition, an ensemble model was constructed using soft voting based on the models created from each of the five cross‐validation sets. The performance of the ensemble model was evaluated using a test dataset. However, our DL model employed a mesh convolutional neural network (MeshCNN) that is innovative and specifically designed for triangular meshes.^19^ MeshCNN leverages the unique properties of meshes for a direct 3D shape analysis and incorporates task‐driven pooling. This pooling approach, in addition to retaining the surface topology, also accesses critical features, making it exceptionally suitable for our study. The training parameters for the MeshCNN model were as follows: batch size of eight, learning rate of 1e‐4, and Adam optimizer. The deep learning model was trained using an NVIDIA GeForce RTX 3090, which provided the necessary computational power and efficiency to handle the complexities and demands of the MeshCNN, ensuring that our model training was both effective and efficient.

### 2.7 Statistical analysis and software

In our study, patient characteristics were meticulously categorized. Continuous variables were expressed as means ± standard deviation or medians with interquartile ranges (IQR), while categorical variables were represented as numbers and percentages (%). To ascertain the differences in continuous variables between the two groups, we used an unpaired t‐test or Mann–Whitney *U* test, depending on the data distribution. Categorical variables, on the other hand, were compared using the Fisher exact test. Statistical significance was set at *p* < .05. 3 To assess our class classification model, we used a range of metrics, including precision, recall, specificity, F1‐score, area under the receiver operating characteristic curve (AUC‐ROC), and Precision‐Recall Area Under the Curve (PR‐AUC). The 95% confidence intervals (CI) for each metric were determined using the bootstrap method with 1000 iterations. This method was used again to compare the metrics of the two models. We then calculated *p*‐values and established 95% confidence intervals through two‐sided tests to determine statistical significance. Python 3.8 was used for base programming, and PyTorch 1.8 as the deep learning library. We used the scikit‐learn package in Python for the output of the metrics, which provided us with robust and reliable statistical analysis capabilities. This combination of advanced programming and deep learning tools was instrumental in effectively executing the training and performance evaluation of the proposed model.

## 3 RESULTS

### 3.1 Patient characteristics

A total of 189 patients who underwent CBA for AF were included during the study period. Table 1 presents the baseline characteristics. The mean age was 65 ± 10 years, and 138 patients (73%) were male. The mean left ventricular ejection fraction was 62% ± 8%. Fifty‐one patients were classified as CBA difficulty group (27%) and 138 (73%) as CBA nondifficulty group.

**TABLE 1:** Patients characteristics.

	All patients (*n* = 189)	CBA nondifficulty group (*n* = 138)	CBA difficulty group (*n* = 51)	*p* value
Age (years)	65 ± 10	66 ± 9	64 ± 13	0.15
Male/female, *n*	138/51	99/39	39/12	0.52
PAF, *n* (%)	178 (94%)	130 (94%)	48 (94%)	0.98
AFL, *n* (%)	40 (21%)	33 (24%)	7 (14%)	0.13
Disease period, (months)	12 [6, 36]	12 [6, 36]	14 [6, 60]	0.47
CHADS2 score	1 [0, 1]	1 [0, 1]	1 [0, 2]	0.04
Hypertension, *n* (%)	93 (49%)	68 (49%)	25 (49%)	0.98
Diabetes mellitus, *n* (%)	23 (12%)	17 (12%)	6 (12%)	0.92
Hemodialysis, *n* (%)	5 (3%)	3 (2%)	2 (4%)	0.51
Creatinine (mg/dL)	0.86 [0.74, 0.98]	0.86 [0.74, 0.97]	0.86 [0.73, 1.01]	0.32
BNP (pg/mL)	40 [20, 89]	36 [22, 69]	53 [15, 94]	0.15
LVEF (%)	62 ± 8	62 ± 7	62 ± 9	0.95
LA Diameter (mm)	38 ± 6	38 ± 5	39 ± 7	0.17

Abbreviations: AFL; atrial flutter, BNP, brain natriuretic peptide; CBA, cryoballoon ablation; LA, left atrium; LVEF, left ventricular ejection fraction; PAF, paroxysmal atrial fibrillation.

### 3.2 Procedural characteristics

**FIGURE 3:**
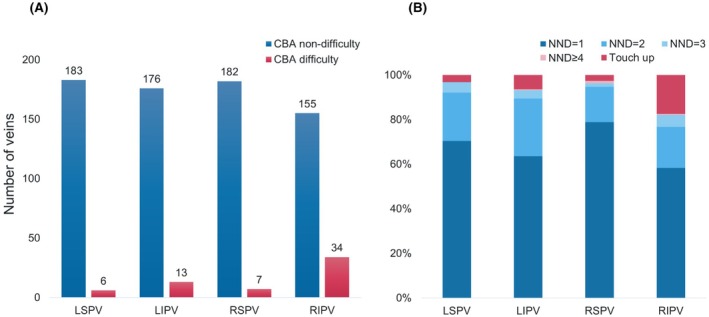
(A) The number of cryoballoon ablation (CBA) difficulty and nondifficulties in each pulmonary vein (B) The number needed to disconnect (NND) and the number of touch‐up ablations in each pulmonary vein. LIPV, left inferior pulmonary vein; LSPV, left superior pulmonary vein; RIPV, right inferior pulmonary vein; RSPV, right superior pulmonary vein.

RIPV presented the greatest difficulty with CBA among the PVs. Out of 189 cases, 34 (18.0%) required either a touch‐up ablation or more than three applications (Figure 3A). The next most difficult PV was the left inferior pulmonary vein (LIPV), with CBA difficulty observed in 13 patients (6.9%). The mean number needed to disconnect (NND) for each PV was as follows: LSPV 1.4 ± 0.6, LIPV 1.4 ± 0.6, right superior pulmonary vein (RSPV) 1.2 ± 0.5, and RIPV 1.5 ± 0.7 (Figure 3B). The procedural outcomes based on operator experience are shown in Table S1. There was no difference in the rate of CBA difficulty between operators with 2–5 years of experience and those with more than 10 years of experience.

### 3.3 Comparison of conventional method versus deep learning model

**FIGURE 4:**
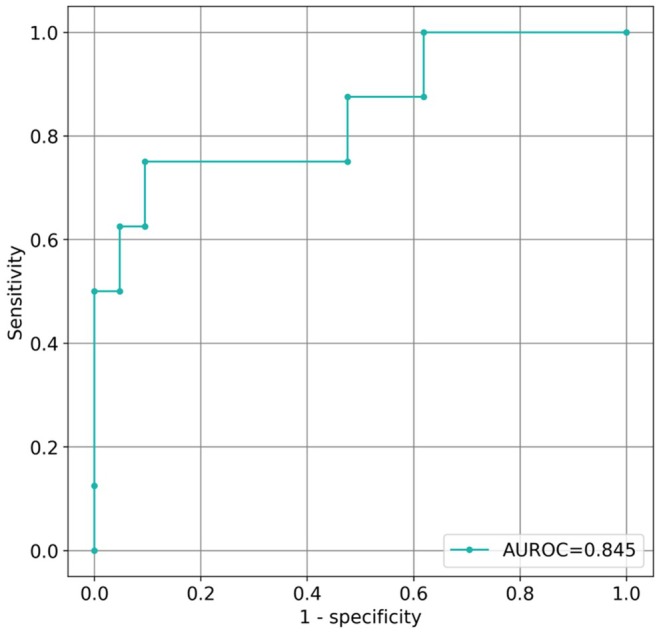
The area under the receiver operating characteristic curve of the deep learning model. The area under the receiver operating characteristic curve (AUROC) value was 0.845.

**FIGURE 5:**
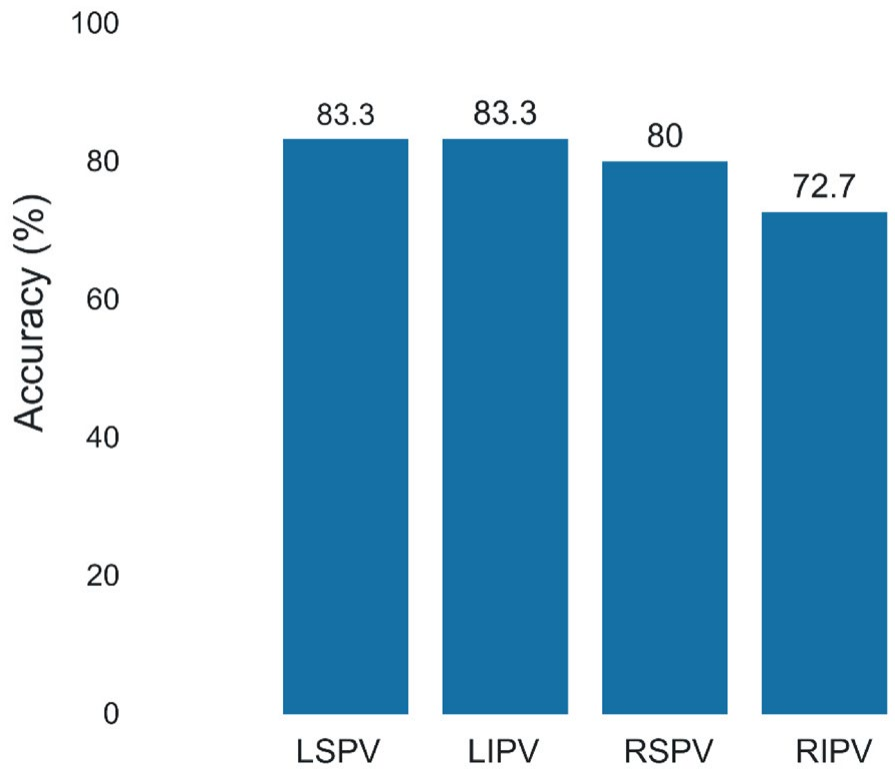
Prediction accuracy of cryoballoon ablation difficulty using a three‐dimensional deep learning model. LIPV, left inferior pulmonary vein; LSPV, left superior pulmonary vein; RIPV, right inferior pulmonary vein; RSPV, right superior pulmonary vein.

Table 2 presents the results of the comparison between the conventional and DL models. The DL model was superior to the conventional method in terms of accuracy (0.793 vs. 0.630, *p* = .042) and specificity (0.796 vs. 0.609, *p* = .022). The AUC‐ROC for the DL model was 0.821 (Figure 4). The prediction accuracy using the DL model for each PV is shown in Figure 5. The prediction accuracy of CBA difficulty was low for right PVs, particularly for the RIPV at 72.7%. The results of the conventional method are shown in Table S2.

**TABLE 2:** Comparison of conventional method versus deep learning model.

	Accuracy	Precision	Recall	Specificity	F1‐score	AUROC	AUPRC
DL model (ensemble)	0.793 (0.655–0.931)	0.586 (0.273–0.900)	0.714 (0.429–1.000)	0.796 (0.636–0.957)	0.622 (0.364–0.880)	0.821 (0.646–0.995)	0.730 (0.468–0.992)
CV set = 0	0.750 (0.625–0.875)	0.500 (0.267–0.714)	0.889 (0.667–1.000)	0.708 (0.545–0.857)	0.636 (0.400–0.813)	0.786 (0.604–0.936)	0.605 (0.272–0.849)
CV set = 1	0.750 (0.625–0.875)	0.538 (0.333–0.750)	0.900 (0.700–1.000)	0.696 (0.542–0.850)	0.667 (0.455–0.833)	0.818 (0.663–0.942)	0.579 (0.314–0.901)
CV set = 2	0.781 (0.656–0.906)	0.600 (0.333–0.857)	0.667 (0.400–0.900)	0.826 (0.684–0.957)	0.625 (0.375–0.824)	0.787 (0.615–0.931)	0.666 (0.346–0.881)
CV set = 3	0.812 (0.688–0.938)	0.643 (0.375–0.889)	0.778 (0.538–1.000)	0.833 (0.696–0.955)	0.700 (0.471–0.880)	0.692 (0.474–0.889)	0.403 (0.207–0.675)
CV set = 4	0.806 (0.677–0.903)	0.600 (0.333–0.875)	0.750 (0.500–1.000)	0.833 (0.680–0.955)	0.667 (0.421–0.857)	0.821 (0.667–0.943)	0.607 (0.263–0.875)
Conventional methods	0.630	0.393	0.686	0.609	0.500	‐	‐
*p*‐value	0.042	0.176	0.680	0.022	0.200	‐	‐

*Note*: The values of each metric for the deep learning model and conventional method are shown along with their differences. Each number represents a metric (upper 95% confidence interval − lower 95% confidence interval). F1 score was calculated as follows: F1‐Score = 2 × Precision × Recall/(Precision + Recall).

Abbreviations: AUPRC, area under the Precision‐Recall Curve; AUROC, area under the receiver operating characteristic curve; CV, cross‐validation; DL, deep learning.

## 4 DISCUSSION

In this study, we developed a 3D DL model to predict CBA difficulty using a polygonal mesh. To the best of our knowledge, this is the first study in the cardiovascular field of DL models created using a polygonal mesh. The main findings of this study are as follows: In contrast to the conventional method of manually measuring CT images, we developed a DL model using a polygonal mesh to predict CBA difficulty, and the current model was able to predict with a higher performance (AUROC = 0.821) than the conventional method.

### 4.1 Prediction accuracy of cryoballoon ablation difficulty using a three‐dimensional deep learning model

In the present study, the RIPV had the most CBA difficulty, followed by the LIPV. This is consistent with previous reports.^4^, ^5^, ^8^, ^9^ Borio et al. reported that the difficulty in occluding the inferior PV during CBA is because of the challenge of aligning the cryoballoon with the PV axis, which is complicated by the branching and angles of the veins.^9^ We also consider that the alignment of the PV axis with the CBA is a key factor contributing to CBA difficulty. However, manually measuring multiple parameters such as branching and angles is challenging. Therefore, we developed a 3D deep learning model that directly learns the anatomical features of the LA and PVs. In this study, we also evaluated the rate of CBA difficulty based on the operator's years of experience. The results showed no significant difference between operators with 2–5 years of experience and those with more than 10 years of experience. This may be because even when the procedures were performed by operators with 2–5 years of experience, they were conducted under the supervision of those with more than 10 years of experience. Although there was no statistically significant difference, operators with over 10 years of experience showed numerically lower rates of CBA difficulty. This suggests that utilizing our 3D DL model may help in selecting the most appropriate operator more accurately. When analyzing cases in which AI predictions were inaccurate, a trend was observed in the RIPV, which exhibited the lowest accuracy rate (72.7%). The RIPV is often the most challenging among the four PVs to occlude and isolate during CBA. In addition to anatomical factors, its proximity to the transseptal puncture site and perturbations induced by phrenic nerve pacing can result in suboptimal balloon‐tissue contact, further reducing the effectiveness of the ablation.^20^ Chierchia et al. investigated the impact of transseptal puncture location on PV occlusion during CBA, reporting that puncturing at the anterior, medial, or posterior aspects of the fossa ovalis did not affect PV occlusion grades, isolation rates, mid‐term outcomes, or complication rates. However, they found that the mean occlusion grade was slightly lower in the RIPV when puncturing in the posterior aspects.^21^ This effect may be particularly relevant in smaller‐bodied Asian populations, where the transseptal puncture site may play a more critical role in achieving effective occlusion. It is challenging to predict the transseptal puncture location and the effects of phrenic nerve pacing based solely on preoperative CT images, which may have contributed to the reduced prediction accuracy in this study.

### 4.2 Optimizing deep learning and data reduction in three‐dimensional structural representations

There are various methods for representing 3D structures, each with its advantages and challenges. Voxel representation provides extensive information about the interior of objects and structures, resulting in excess extraneous data.^13^, ^14^, ^15^ Consequently, a larger number of cases is required for an effective analysis. In contrast, point clouds and polygon meshes offer only surface information, resulting in less data compared with the voxel method.^13^ In addition, point clouds struggle to determine whether the adjacent points belong to different structures. Polygon meshes, however, can differentiate between these because of their continuous surface representation, making them particularly suitable for representing complex structures such as the heart and its adjacent components, including the pulmonary veins, left atrium, and left atrial appendage.

Polygonal meshes consist of vertices, edges, and faces, and even with edge collapse, which reduces the number of vertices, the structure remains intact. This reduction in information volume can enhance AI learning, especially with high‐dimensional data such as images. The dataset used in this study was relatively small; however, this reduction likely facilitated the learning process. In this study, we utilized preoperative CT images; however, this method can also be applied to magnetic resonance imaging (MRI) and 3D electroanatomical mapping. In the EP field, preoperative CT and MRI images are commonly performed, and intraoperative 3D electroanatomical mapping is often acquired. Applying this method could enable more efficient image‐based learning in the EP field. In an AI analysis, having more cases generally leads to better predictions. However, collecting a substantial number of cases from a single facility is challenging and requires collaboration among multiple facilities. Consequently, this approach faces several challenges, including privacy concerns, data format inconsistencies, and procedural differences.

### 4.3 Clinical implications

A 3D DL model based on preoperative cardiac CT images is useful for identifying patients who are anatomically predicted to experience difficulties with CBA. In such cases, a detailed reevaluation of the CT images or optimization of the transseptal puncture site may help overcome procedural challenges. Moreover, selecting a more experienced operator and proceeding with RFCA may contribute to reducing procedure time, cost, and radiation exposure. Conversely, this model may also assist in identifying patients suitable for safe and effective CBA even when performed by less experienced operators, thereby enhancing training opportunities while maintaining procedural safety and efficacy.

### 4.4 Limitations

The study was based on data from a single institution with a limited number of cases, leading to wide confidence intervals (95% CI) in model comparison analyses, complicating the detection of significant differences. Additionally, multicenter validation was not performed. Incorporating data from multiple institutions in future studies will be valuable for further validating the model's robustness and ensuring its broader applicability. Because the institution also serves as a training facility, there is a relatively high rate of touch‐up procedures, suggesting that variability in operator skill levels may have influenced the outcomes.

## 5 CONCLUSION

We developed a 3D deep learning model that can detect difficulties in CBA using a polygonal mesh representation. By predicting difficult cases in advance, strategies can be developed to increase success rates.

## AUTHOR CONTRIBUTIONS

Kazutaka Nakasone: concept/clinical practice/interpretation/data sampling/drafting article. Makoto Nishimori: design/data analysis/drafting article/ approval of article. Masakazu Shinohara: data collection/statics. Kunihiko Kiuchi: data collection/statics. Mitsuru Takami: data collection/statics. Kimitake Imamura: data collection/statics. Yu Izawa: data collection/statics. Toshihiro Nakamura: data collection/statics. Yusuke Sonoda: data collection/statics. Hiroyuki Takahara: data collection/statics. Kyoko Yamamoto: data collection/statistics. Yuya Suzuki: data collection/statics. Kenichi Tani: data collection/statics. Hidehiro Iwai: data collection/statics. Yusuke Nakanishi: data collection/statistics. Ken‐ichi Hirata: approval of article. Koji Fukuzawa: approval of article.

## FUNDING INFORMATION

There are no sources of funding related to this study.

## CONFLICT OF INTEREST STATEMENT

The section of arrhythmia is supported by endowments from Abbott Japan, Boston Scientific Japan, and Medtronic Japan. Ken‐ichi Hirata chairs the section, and Koji Fukuzawa and Kimitake Imamura belong to the Section. Koji Fukuzawa has received a scholarship donation from Biotronik Japan. However, all authors report no conflict of interest for this manuscript's contents. Ken‐ichi Hirata is a member of Circulation Journal's Editorial Team.

## ETHICS STATEMENT

The study protocol was approved by the institutional ethics committee. All patients provided written informed consent. Name of the ethics committee: Kobe University Medical Ethical Committee. Reference number: No. B220174.

## PATIENT CONSENT STATEMENT

Written informed consent was obtained from the patients.

## Supporting information


Data S1.

## Data Availability

The data, analytic methods, and study materials will not be made available to other researchers for purposes of reproducing the results or replicating the procedure.

## References

[1] Joglar JA , Chung MK , Armbruster AL , Benjamin EJ , Chyou JY , Cronin EM , et al. 2023 ACC/AHA/ACCP/HRS guideline for the diagnosis and management of atrial fibrillation: a report of the American College of Cardiology/American Heart Association joint committee on clinical practice guidelines. Circulation. 2024;149:e1–e156. 38033089 10.1161/CIR.0000000000001193 PMC11095842

[2] Squara F , Zhao A , Marijon E , Latcu DG , Providencia R , Di Giovanni G , et al. Comparison between radiofrequency with contact force‐sensing and second‐generation cryoballoon for paroxysmal atrial fibrillation catheter ablation: a multicentre European evaluation. EP Europace. 2015;17:718–724. 10.1093/europace/euv060 25840289

[3] Kuck K‐H , Brugada J , Fürnkranz A , Metzner A , Ouyang F , Chun KJ , et al. Cryoballoon or radiofrequency ablation for paroxysmal atrial fibrillation. N Engl J Med. 2016;374:2235–2245. 27042964 10.1056/NEJMoa1602014

[4] Yasuoka R , Kurita T , Kotake Y , Hashiguchi N , Motoki K , Kobuke K , et al. Particular morphology of inferior pulmonary veins and difficulty of cryoballoon ablation in patients with paroxysmal atrial fibrillation. Circ J. 2017;81:668–674. 28216515 10.1253/circj.CJ-16-1161

[5] Kajiyama T , Miyazaki S , Matsuda J , Watanabe T , Niida T , Takagi T , et al. Anatomic parameters predicting procedural difficulty and balloon temperature predicting successful applications in individual pulmonary veins during 28‐mm second‐generation cryoballoon ablation. JACC: Clin Electrophysiol. 2017;3:580–588. 29759431 10.1016/j.jacep.2017.01.004

[6] Matsumoto Y , Muraoka Y , Funama Y , Mito S , Masuda T , Sato T , et al. Analysis of the anatomical features of pulmonary veins on pre‐procedural cardiac CT images resulting in incomplete cryoballoon ablation for atrial fibrillation. J Cardiovasc Comput Tomogr. 2019;13:118–127. 30466810 10.1016/j.jcct.2018.11.005

[7] Ströker E , de Asmundis C , Saitoh Y , Velagić V , Mugnai G , Irfan G , et al. Anatomic predictors of phrenic nerve injury in the setting of pulmonary vein isolation using the 28‐mm second‐generation cryoballoon. Heart Rhythm. 2016;13:342–351. 26573972 10.1016/j.hrthm.2015.10.017

[8] Chen X , Fang P , Liu Z , He J , Tang M , Liu J , et al. Pulmonary vein anatomy is associated with cryo kinetics during cryoballoon ablation for atrial fibrillation. Arq Bras Cardiol. 2018;110:440–448. 29898044 10.5935/abc.20180071 PMC5967138

[9] Borio G , Maj R , Alessandro R , Stroker E , Sieira J , Osorio TG , et al. Pulmonary veins anatomical determinants of cooling kinetics during second‐generation cryoballoon ablation. J Cardiovasc Electrophysiol. 2020;31:629–637. 31943519 10.1111/jce.14356

[10] Hayashi T , Murakami M , Saito S , Iwasaki K . Characteristics of anatomical difficulty for cryoballoon ablation: insights from CT. Open Heart. 2022;9:e001724. 34992156 10.1136/openhrt-2021-001724 PMC8739445

[11] Chen L , Huang S‐H , Wang T‐H , Lan T‐Y , Tseng VS , Tsao H‐M , et al. Deep learning‐based automatic left atrial appendage filling defects assessment on cardiac computed tomography for clinical and subclinical atrial fibrillation patients. Heliyon. 2023;9(1):e12945. 10.1016/j.heliyon.2023.e12945 36699283 PMC9868534

[12] Liu C‐M , Chang S‐L , Chen H‐H , Chen W‐S , Lin Y‐J , Lo L‐W , et al. The clinical application of the deep learning technique for predicting trigger origins in patients with paroxysmal atrial fibrillation with catheter ablation. Circ Arrhythm Electrophysiol. 2020;13:e008518. 33021404 10.1161/CIRCEP.120.008518

[13] Gebhardt S , Payzer E , Salemann L , Fettinger A , Rotenberg E , Seher C . Polygons, point‐clouds and voxels: a comparison of high‐fidelity terrain representations. Paper presented at: Simulation interoperability workshop and special workshop on reuse of environmental data for simulation—processes, standards, and lessons learned. 2009.

[14] Hu J , Qing Z , Liu R , Zhang X , Lv P , Wang M , et al. Deep learning‐based classification and voxel‐based visualization of frontotemporal dementia and Alzheimer's disease. Front Neurosci. 2021;14:626154. 33551735 10.3389/fnins.2020.626154 PMC7858673

[15] Singh SP , Wang L , Gupta S , Goli H , Padmanabhan P , Gulyás B . 3D deep learning on medical images: a review. Sensors. 2020;20:5097. 32906819 10.3390/s20185097 PMC7570704

[16] Akita T , Kiuchi K , Fukuzawa K , Shimane A , Matsuyama S , Takami M , et al. Lesion distribution after cryoballoon ablation and hotballoon ablation: late‐gadolinium enhancement magnetic resonance imaging analysis. J Cardiovasc Electrophysiol. 2019;30:1830–1840. 31310389 10.1111/jce.14073

[17] Fedorov A , Beichel R , Kalpathy‐Cramer J , Finet J , Fillion‐Robin J‐C , Pujol S , et al. 3D slicer as an image computing platform for the quantitative imaging network. Magn Reson Imaging. 2012;30:1323–1341. 22770690 10.1016/j.mri.2012.05.001 PMC3466397

[18] Alessandro Muntoni J , Cignoni P , Luaces A , Scott R , Luzpaz OZ , Zhang F . cnr‐isti‐vclab/PyMeshLab: PyMeshLab v2023.12.post1 (v2023.12.post1). *Zenodo*. 2024 10.5281/zenodo.10573055

[19] Hanocka R , Hertz A , Fish N , Giryes R , Fleishman S , Cohen‐Or D . MeshCNN: a network with an edge. ACM Trans Graph. 2019;38(4):1–12. 10.1145/3306346.3322959

[20] Martins RP , Nicolas A , Galand V , Pichard C , Behar N , Chérel C , et al. The challenging right inferior pulmonary vein: a systematic approach for successful cryoballoon ablation. Arch Cardiovasc Dis. 2019;112:502–511. 31447317 10.1016/j.acvd.2019.05.006

[21] Chierchia GB , Casado‐Arroyo R , de Asmundis C , Rodriguez‐Manero M , Sarkozy A , Conte G , et al. Impact of transseptal puncture site on acute and mid‐term outcomes during cryoballoon ablation: a comparison between anterior, medial and posterior transatrial access. Int J Cardiol. 2013;168:4098–4102. 23928339 10.1016/j.ijcard.2013.07.079

