# Supplementary material for: Prediction of difficulty in cryoballoon ablation with a three‐dimensional deep learning model using polygonal mesh representation
Source: J Arrhythm. 2025 Apr 25;41(2):e70078. doi: 10.1002/joa3.70078 (PMC12022427; doi:10.1002/joa3.70078)
Supplement: Supplementary file 1 — Data S1. [file JOA3-41-e70078-s001.docx]

**Supplemental Table 1**

|  | All patients  (n = 189) | 2–5 years operators  (n = 125) | > 10 years operators  (n = 64) | p value |
| --- | --- | --- | --- | --- |
| CBA difficulty, n (%) | 51 (27.0 %) | 38 (30.4 %) | 13 (20.3 %) | 0.14 |
| LSPV, n (%) | 6 (3.2 %) | 5 (4.0 %) | 1 (1.6 %) | 0.37 |
| LIPV, n (%) | 13 (6.9 %) | 9 (7.2 %) | 4 (6.3 %) | 0.81 |
| RSPV, n (%) | 7 (3.7 %) | 6 (4.8 %) | 1 (1.6 %) | 0.26 |
| RIPV, n (%) | 34 (18.0 %) | 25 (20.0 %) | 9 (14.1 %) | 0.31 |

**Supplemental Table 1:** **The procedural outcomes based on operator experience.** CBA, cryoballoon ablation; LIPV; left inferior pulmonary vein, LSPV; left superior pulmonary vein, RIPV; right inferior pulmonary vein, RSPV; right superior pulmonary vein.

**Supplemental Table 2**

|  | **Yes** | **No** |
| --- | --- | --- |
| Left lateral ridge < 4.7mm | 8 | 181 |
| LSPV ovality > 50.5% | 1 | 188 |
| LSPV ostium-bifurcation distance > 26.1mm | 67 | 122 |
| RIPV ostium-bifurcation distance < 10.4mm | 27 | 162 |
| If any of the above conditions is satisfied | 89 | 100 |

**Supplemental Table 2: The results of the conventional method.** LSPV, left superior pulmonary vein; RIPV, right inferior pulmonary vein.
